# Ericoid mycorrhizal root fungi and their multicopper oxidases from a temperate forest shrub

**DOI:** 10.1002/ece3.67

**Published:** 2012-01

**Authors:** Nina Wurzburger, Brian P Higgins, Ronald L Hendrick

**Affiliations:** 1 Odum School of Ecology, University of GeorgiaAthens, Georgia; 2 Department of Microbiology, University of GeorgiaAthens, Georgia; 3 Georgia Health Sciences University - University of Georgia Medical PartnershipAthens, Georgia; 4 School of Environment and Natural Resources, The Ohio State UniversityColumbus, Ohio

**Keywords:** Ericoid mycorrhizal fungi, ITS rDNA, Polyphenol oxidase, Rhododendron

## Abstract

Ericoid mycorrhizal fungi (ERM) may specialize in capturing nutrients from their host's litter as a strategy for regulating nutrient cycles in terrestrial ecosystems. In spite of their potential significance, we know little about the structure of ERM fungal communities and the genetic basis of their saprotrophic traits (e.g., genes encoding extracellular enzymes). *Rhododendron maximum* is a model ERM understory shrub that influences the nutrient cycles of montane hardwood forests in the southern Appalachians (North Carolina, USA). We sampled ERM roots of *R. maximum* from organic and mineral soil horizons and identified root fungi by amplifying and sequencing internal transcribed spacer (ITS) ribosomal DNA (rDNA) collected from cultures and clones. We observed 71 fungal taxa on ERM roots, including known symbionts *Rhizoscyphus ericae* and *Oidiodendron maius*, putative symbionts from the Helotiales, Chaetothyriales, and Sebacinales, ectomycorrhizal symbionts, and saprotrophs. Supporting the idea that ERM fungi are adept saprotrophs, richness of root-fungi was greater in organic than in mineral soil horizons. To study the genetic diversity of oxidative enzymes that contribute to decomposition, we amplified and sequenced a portion of genes encoding multicopper oxidases (MCOs) from ERM ascomycetes. Most fungi possessed multiple copies of MCO sequences with strong similarities to known ferroxidases and laccases. Our findings indicate that *R. maximum* associates with a taxonomically and ecologically diverse fungal community. The study of MCO gene diversity and expression may be useful for understanding how ERM root fungi regulate the cycling of nutrients between the host plant and the soil environment.

## Introduction

Soil microorganisms are largely responsible for the recycling of nutrients within terrestrial ecosystems (van der Heijden et al. 2008). Root-associating fungi such as mycorrhizal symbionts can acquire nutrients directly from soil organic matter yet they vary widely in their ability to do so (Read et al. 2004). Emergent patterns among the types of mycorrhizal associations suggest a functional relationship between the leaf litter traits of plant hosts and the nutrient-acquiring traits of their fungal symbionts. Across the mycorrhizal types arbuscular mycorrhizas (AM), ectomycorrhizas (ECM), and ericoid mycorrhizas (ERM), there is a decline in the decomposability of leaf litter from the plant host (Cornelissen et al. 2001) and a concomitant increase in the ability of the host's mycorrhizal symbionts to obtain nutrients from organic substrates (i.e., saprotrophy) (Read et al. 2004 and references therein). These patterns suggest that mycorrhizal fungi facilitate nutrient feedbacks between plants and soils by specializing in nutrient acquisition from the soils promoted by their host.

ERM plants operate at one end of this functional spectrum. Ericaceous plants produce polyphenol-rich leaf litter that leads to accumulations of organic matter and recalcitrant forms nitrogen (N) in soils (Read et al. 2004). These leaf litter traits are complemented by the saprotrophic nature of ERM fungi. In pure culture, ERM fungi produce a broad suite of extracellular enzymes (e.g., phosphatases, proteases, cellulases, and polyphenol oxidases) that contribute to decomposition and nutrient acquisition (Read et al. 2004). Of this group, polyphenol oxidases may be of particular significance, as they can facilitate the release of complexed-N from the organic matter that accumulates under ERM hosts (Bending and Read 1996, 1997). Although ERM hosts are distributed worldwide (Kron and Lutyen 2005), they often persist in ecosystems dominated by ECM and AM hosts (i.e., temperate and boreal forests; Read et al. 2004). If ERM hosts create pools of soil nutrients that are accessible to their own fungal symbionts but are less available to the AM and ECM fungi of neighboring plants, the mechanisms behind this are central to our knowledge of nutrient partitioning and biogeochemical cycling. In spite of the intriguing relationship between the traits of ERM host leaf litter and ERM fungi, we lack specific knowledge about the fungal assemblages on ERM roots and how these fungi acquire nutrients from organic matter.

The identities of ERM fungi have remained enigmatic for a variety of reasons. Not only are ERM fungi challenging to identify because they lack conspicuous fruiting bodies, but also these fungi are finely distributed within hair roots and are not universally culturable (Perotto et al. 2002; Allen et al. 2003). *Oidiodendron maius* and members of the *Rhizoscyphus ericae* complex are frequently cultured from roots of ERM hosts; however, direct molecular evidence indicates that these fungi are not dominants of ERM fungal communities (Allen et al. 2003; Bougoure and Cairney 2005a). In fact, taxa from the Sebacinales were the most frequently cloned fungi from ERM roots of *Gaultheria shallon* (Allen et al. 2003) and were ubiquitous in roots of ericaceous shrubs across the globe (Selosse et al. 2007). Several unknown fungi have been repeatedly observed on ERM roots (Bougoure and Cairney 2005a, b) leading to questions about the functional role of these diverse root fungi.

Direct evidence for how ERM fungi promote nutrient acquisition from organic matter is also limited. The ability of ERM fungi to oxidize polyphenols may be a key mechanism for releasing N from the polyphenol-rich organic matter that accumulates under ERM hosts. Polyphenol oxidases or multicopper oxidases (MCOs) are a diverse group of enzymes including laccases, L-ascorbate oxidases, and ferroxidases (Hoegger et al. 2006). Only the two well-studied species of ERM fungi (*O. maius* and *R. ericae*) have been tested to produce MCOs in pure culture (Bending and Read 1997) and there is no genetic information about MCOs from ERM fungi. Of MCOs, laccases may be particularly important for the decomposition of organic matter (Lyons et al. 2003; Kellner et al. 2007). The abundance of MCO genes and their diversity among ERM root-fungi could provide a valuable genetic perspective about how ERM fungi contribute to decomposition processes and nutrient acquisition for their hosts (Read and Perez–Moreno 2003).

Here, we examine fungi and their MCO genes from ERM roots of *Rhododendron maximum* in a southern Appalachian hardwood forest. *Rhododendron maximum* is a model ERM plant species because it is common in the forest understory (Monk et al. 1985), produces recalcitrant leaf litter (Hunter et al. 2003), and has a marked influence on plant community composition (Baker and Van Lear 1998; Lambers and Clark 2003) and patterns of soil carbon and N cycling (Boettcher and Kalisz 1990; Wurzburger and Hendrick 2007). We previously demonstrated that ERM roots of *R. maximum*can acquire more of the recalcitrant N derived from its leaf litter than can ECM and AM roots of neighboring plant species (Wurzburger and Hendrick 2009). We have also observed elevated activities of extracellular MCOs in soils under *R. maximum*, suggesting that ERM fungi are producing these enzymes and directly contributing to decomposition (Wurzburger and Hendrick 2007). These observations lead to questions about the fungal assemblages on ERM roots of *R. maximum* and the diversity of MCO genes among these fungi.

We first sought to document the composition of fungi associating with ERM roots of *R. maximum* and how the fungal community differs between organic and mineral soil horizons. Since southern Appalachian forests are remarkably species-rich in plants (Hardt and Swank 1997) and ECM fungi (Walker et al. 2005), we hypothesized that a taxonomically rich ERM fungal community associates with *R. maximum*. Because of the saprotrophic nature of ERM fungi, we hypothesized that the fungal richness on ERM roots would be greater in organic than in mineral soil horizons. Our second objective was to characterize MCO gene sequences from the fungi we cultured from ERM roots. We used fungal cultures to screen for MCO genes, allowing us to link the identity of the fungus to MCO gene sequences. Since we previously documented elevated extracellular MCO activities in *R. maximum* soils (Wurzburger and Hendrick 2007), we hypothesized that ERM fungi would possess multiple copies of MCOs, including a number of putative laccases.

## Materials and Methods

We collected root samples in four (4 × 4 m^2^) replicate plots in *R. maximum* L. thickets of mature hardwood forests along a high elevation (c.1450 m) ridge in Coweeta Hydrologic Lab and Nantahala National Forest, North Carolina, USA. *Rhododendron maximum* is an evergreen shrub with sclerophyllous foliage that forms dense thickets (up to 34 Mg/ha in aboveground biomass; Baker and Van Lear 1998) that spread by layering and root sprouts (Monk et al. 1985). These forests are characterized by northern hardwood species *Quercus rubra* L., *Betula lenta* L., *B. alleghaniensis* Britt., *Acer rubrum* L., and *Fraxinus americana* L. Soils are inceptisols derived from igneous and metamorphic rock. The average annual temperature is 9.4°C and the average annual precipitation is 250 cm (Swift et al. 1988).

### Culturing fungi from ERM roots

In order to characterize the broad assemblage of fungi colonizing ERM roots, we cultured and cloned root fungi from ERM roots. Pure cultures of ERM root-fungi also provided a means to screen individual genomes for MCO genes. We collected *R. maximum* ERM hair roots from the O horizon from each plot in both spring and late summer. Hair roots were cleaned under a dissecting microscope, inspected for colonization of fungi, and rinsed with sterile water. From each sample, 20–30 1-cm root sections, (222 root sections in total), were surface sterilized, plated on potato dextrose agar, and maintained in the dark at 19°C (Allen et al. 2003). We discarded cultures dominated by rapidly growing, sporulating fungi. Genomic DNA was extracted from each fungal culture using Qiagen DNeasy Plant Mini Kit (Valencia, CA). The ribosomal DNA (rDNA) internal transcribed spacer (ITS) region (ITS1, 5.8S rDNA gene, and ITS2) was amplified from each DNA preparation with fungal specific primers ITS4 and ITS1-F (White et al. 1990; Gardes and Bruns 1993). In order to pare down the number of samples to sequence, ITS products were typed by restriction fragment length polymorphisms (RFLP) using HinfI and AluI (New England Biolabs, Ipswich, MA), and amplified products from each RFLP type were sequenced in both directions.

### Cloning fungal ITS products from ERM roots

In late summer, we randomly sampled three 10-cm deep and 2-cm-diameter soil cores from each plot under thickets of *R. maximum*. The distinctive nature of ERM hair roots (<0.1-mm diameter) relative to fine roots of ECM and AM roots of canopy trees (0.1- to 0.5-mm diameter) provides a visual means to sort ERM hair roots of *R. maximum* from other plant species. In addition, herbaceous plant species, such as grasses, are lacking from thickets of *R. maximum*. For further verification, a subset of root samples was verified for ERM root structure (the lack of root hairs and the presence of hyphal coils in root cortical cells) under a compound microscope. Under a dissecting microscope, hair roots were cleaned of soil and organic material using a fine brush and rinsed with sterile deionized water. A root sample consisted of 10–20 root fragments (1 cm in length) from the O and A horizon of each core. After lyophilizing roots and extracting DNA, we amplified the rDNA and ITS region (as above). ITS products were visualized on 2% agarose gels, purified with Column-Pure DNA Gel Recovery kits (Lamda Biotech, St. Louis, MO), and cloned into the pCR2.1 vector with the TOPO TA Cloning Kit (Invitrogen, Carlsbad, CA). From each sample, we purified plasmid DNA from 20–30 *Escherichia coli* colonies using lithium chloride minipreps (Ausubel et al. 1998). Plasmid inserts were amplified using M13For/Rev primers, and RFLP-typed using HinfI and AluI and one clone per RFLP type per core was sequenced. Plasmid DNA from each RFLP-type was purified using Qiagen Plasmid miniprep purification kits and sequenced in both directions. Sequences were aligned using SeqMan software (DNASTAR Madison, WI) and small subunit (SSU) Group I introns (Perotto et al. 2000) were removed. Sequences with at least 97% identity were assigned to the same sequence type (O’Brien et al. 2005). BLAST nucleotide searches (Altschul et al. 1997) against the GenBank nucleotide database (http://blast.ncbi.nlm.nih.gov/Blast.cgi) were conducted to determine taxonomic affinities. The fungal class that captured all the top BLAST hits was used as a putative classification for each fungal taxon. Taxa were classified to phylum if the 5.8s and at least half of the ITS1 or ITS2 regions provided similarity with known sequences. In several cases, unknown, uncultured fungi dominated the BLAST hits making classification problematic. Chimeric sequences, an artifact of cloning DNA from environmental samples (O’Brien et al. 2005), were identified when the ITS1 and ITS2 regions differed in their taxonomic identity and excluded from the analysis.

### Fungal community structure and richness

We estimated fungal richness in the O and A horizons using EsimateS software from the frequency of cloned ITS sequences from each replicate sampling plot (*n* = 4) (Colwell 2005). We produced taxa accumulation curves, using plot-level data as the experimental unit (1000 bootstrap replicates with replacement using the Chao1 richness estimator [Chao 2004]). The relative frequency of each fungal taxon observed in culture and in each the O and A soil horizon was calculated on a plot-level basis and averaged across the four plots.

### MCO gene sequences

To explore the genetic diversity of MCO genes from individual fungal genomes, we amplified DNA from cultures of ERM root fungi, which consisted of ascomycetes. We amplified the region between copper-binding domains II and III (*lcc2*) of MCOs from genomic DNA of cultured fungi using the degenerate primers LAC3FOR and LAC4REV (Lyons et al. 2003). PCR products were visualized on 2% agarose gels and bands (0.9–1 kb) were purified and cloned (as above). Plasmid DNA from 10 positive clones was purified, amplified, and RFLP-typed (as above). Clones exhibiting unique RFLP patterns from each isolate were sequenced, and the results submitted for BLASTX analysis (Altschul et al. 1997). We retained nucleotide sequences with similarity to known MCOs (or hypothetical proteins). Sequences from our study that were less than 97% identical to each other were considered unique types. For each sequence we determined the correct reading frame and removed introns with Augustus gene prediction software (Stanke et al. 2004). Amino acid sequences were entered into a BLASTP search in Genbank. MCO protein sequences were aligned with 70 additional ascomycetous and basidiomycetous MCO sequences using CLUSTALX (BLOSUM62 cost matrix; [Larkin et al. 2007]). A maximum likelihood tree was constructed using PhyML (WAG substitution model; 1000 bootstrap replicates; Guindon and Gascuel 2003).

## Results

### Fungal ITS types

ERM roots of *R. maximum* support a rich fungal community; we observed 71 unique ITS types from our culture and clone collection. The observed fungi were taxonomically diverse and included members of the Ascomycota (47), Basidiomycota (14), Zygomycota (1), Chytridiomycota (1), or had an unknown identity (8). From 222 plated ERM root segments, we isolated 52 cultures of fungi, representing 17 unique ITS fungal taxa (Table 1). We identified 57 unique ITS fungal taxa from 352 screened fungal ITS clones (Table 1). Of 12 soil cores of the O and A horizon, we successfully amplified fungal DNA from 11 root samples from the O horizon, and although nine of 12 soil cores possessed ERM roots in the A horizon, only five of those samples provided us with amplified DNA. On average, we screened 25 clones per root sample from which we obtained an average of 20 fungal ITS products. The remaining clones contained no plasmid insert, an *R. maximum* ITS insert, or provided no sequence data after repeated attempts. We observed three cloned fungal ITS types among the cultured fungi. Four chimeric sequences were excluded from our analysis. Eight sequences among the 71 possessed poor matches to those in Genbank could not be assigned to a phylum or reliably screened for chimeras. ITS sequences are available in Genbank under accession numbers HM030566–HM030635.

**Table 1:** Internal transcribed spacer (ITS) sequence types, classification, and closest BLAST match of fungi sampled from ERM roots of *Rhododendron maximum*. Relative frequencies (mean of four sampling plots) of fungal taxa expressed from clone samples from the O and A soil horizons and root cultures from the O horizon. ITS sequences are available in Genbank under accession numbers HM030566–HM030635

						Relative frequency (%)		
						
taxon	Putative classification	Closest BLAST match with known taxon	*E*-value	Identity (%)	Overlap (bp)	O horizon clone	A horizon clone	Culture
Ascomycota								
c6	Eurotiomycetes	*Capronia* sp. UBCTRA1522.6 (AF284126)	0	98	377	12.7	0.9	0
c2	Eurotiomycetes	*Capronia* sp. UBCTRA 1322.11 (AF284128)	0	98	377	11.1	1.5	0
c32	Eurotiomycetes	*Oidiodendron maius* strain UAMH 8921 (AF062800)	0	99	525	4.7	3.8	46.0
c42	Eurotiomycetes	*Capronia* sp. 96003a (EU139159)	2e^−173^	86	513	0.7	1.0	0
c52	Eurotiomycetes	*Elaphomyces decipiens* voucher Trappe 12436 (EU837229)	0	94	632	0.4	0	0
c50	Eurotiomycetes	*Capronia* sp. UBCTRA 1322.11 (AF284128)	0	98	375	0.3	0	0
c1	Leotiomycetes	*Rhizoscyphus ericae* (AM887700)	0	93	545	12.3	15.6	0
c15	Leotiomycetes	*Neofabraea malicortici*s (AF141189)	0	89	542	4.4	0	0
c26	Leotiomycetes	*Rhizoscyphus ericae* (AM887700)	4e^−164^	85	507	2.1	0	0
c74	Leotiomycetes	*Phialocephala fortinii* isolate PFO-3 (EF093159)	4e^−160^	82	600	2.0	16.7	0
c18	Leotiomycetes	*Alatospora acuminata* strain ccm-F12186 (AY204588)	0	88	516	1.2	0	0
c10	Leotiomycetes	*Rhizoscyphus ericae* (EU221877)	0	88	793	0.9	0	0
c40	Leotiomycetes	*Rhizoscyphus ericae* (EU221877)	0	91	514	0.9	0	0
c7	Leotiomycetes	*Phialophora finlandia* strain CBS 444.86 (AF486119)	0	88	780	0.4	0	2.0
c14	Leotiomycetes	*Dermea viburni* (AF141163)	0	96	533	0.3	0.5	10.0
c19	Leotiomycetes	*Arachnopeziza aurata* (U57496)	0	95	542	0.3	0	0
c59	Leotiomycetes	*Arachnopeziza aurata* (U57496)	0	91	516	0.3	0	0
c9	Leotiomycetes	*Rhizoscyphus ericae* (EU221877)	8	97	857	0	10.4	0
c16	Leotiomycetes	*Articulospora tetracladia* strain F-03680c (EU998926)	1e^−159^	84	514	0	17.7	0
c80	Leotiomycetes	*Cryptosporiopsis ericae* isolate LLD-13–38a (EF413595)	0	98	480	0	0	11.0
c81	Leotiomycetes	*Articulospora tetracladia* strain F-4494 (EU998918)	0	97	517	0	0	1.0
c82	Leotiomycetes	*Phialophora finlandia* strain CBS 444.86 (AF486119)	0	92	457	0	0	2.0
c83	Leotiomycetes	*Fulvoflamma eucalypti* strain CPC 11243 (DQ195779)	0	99	463	0	0	1.0
c86	Leotiomycetes	*Pilidium acerinum* voucher BPI 843555 (AY487091)	0	97	440	0	0	3.0
c24	Sordariomycetes	*Pochonia bulbillosa* strain 38G272 (EU999952)	0	100	569	3.3	1.4	0
c48	Sordariomycetes	*Chaetosphaeria chloroconia* (AF178542)	0	96	511	1.1	0	0
c30	Sordariomycetes	*Metarhizium flavoviride* strain NHJ6171 (AY646392)	0	85	574	0.9	0	0
c23	Sordariomycetes	*Cordyceps ophioglossoides* strain CCRC 32220 (AY245636)	0	91	521	0.4	0	0
c58	Sordariomycetes	*Cephalotheca foveolata* (AB278171)	0	89	548	0.4	0	0
c35	Sordariomycetes	*Hypocrea koningii* (AJ301990)	0	99	638	0.4	0	0
c57	Sordariomycetes	*Hypoxylon perforatum* isolate M4 (FJ464593)	0	96	583	0	0	3.0
c17	Sordariomycetes	*Phialophora* sp. aurim712 (DQ069046)	0	95	494	0	0.5	0
c60	Sordariomycetes	*Chloridium virescens* strain ICMP15193 (EF029220)	0	95	517	0	0	2.0
c61	Sordariomycetes	CSP279468 *Chaetomium* sp. 6/97–38 (AJ279468)	0	94	495	0	0	3.0
c62	Sordariomycetes	*Cephalotheca sulfurea* (AB278194)	4e^−129^	81	506	0	1.0	0
c63	Sordariomycetes	*Xylaria persicaria* strain F-165174 (AY909022)	0	99	500	0	0	3.0
c84	Sordariomycetes	*Monochaetia camelliae* (AF377286)	0	96	558	0	0	3.0
c85	Sordariomycetes	*Glomerella acutata* strain DAOM214992 (EU400154)	0	100	562	0	0	1.0
c88	Sordariomycetes	*Ophiostoma dentifundum* strain CMW13016 (AY495434)	0	100	535	0	0	1.0
c41	Dothideomycetes	*Ramichloridium cerophilum* strain CBS 103.59 (EU041798)	2e^−162^	85	511	0.9	0	0
c44	Dothideomycetes	*Cenococcum geophilum* isolate cl2.19 (AY394919)	0	99	531	0.9	0	0
c27	Dothideomycetes	*Mycosphaerella parkiiaffinis* strain CBS 120737 (EF394846)	1e^−179^	87	516	0.4	0	0
c38	Dothideomycetes	*Trimmatostroma cordae* (AJ244263)	1e^−139^	82	522	0.3	0	0
c79	Dothideomycetes	*Coleophoma empetri* isolate 38 (FJ480134)	0	100	524	0	0	2.0
c87	Dothideomycetes	*Massarina corticola* (AF383957)	0	97	475	0	0	2.0
c11	Lecanoromycetes	*Furcaspora eucalypti* strain CBS 119111 (EF110613)	3e^−152^	84	491	1.5	0	0
c28	Lecanoromycetes	*Sarea* sp. BC16 (DQ317349)	2e^−148^	86	454	0.4	0	0
Basidiomycota								
c13	Hymenomycetes	*Sebacina vermifera* AFTOL-ID 1877 (DQ520096)	4e^−120^	83	417	13.5	1.5	0
c21	Hymenomycetes	*Sebacina vermifera* AFTOL-ID 1877 (DQ520096)	4e^−170^	83	594	3.3	1.9	0
c25	Hymenomycetes	*Sebacina vermifera* AFTOL-ID 1877 (DQ520096)	2e^−162^	82	596	1.7	0	0
c34	Hymenomycetes	*Sebacina vermifera* AFTOL-ID 1877 (DQ520096)	2e^−129^	85	570	0.4	2.7	0
c51	Agaricomycetes	*Scleroderma citrinum* isolate AWW212 (EU718119)	0	99	663	5.3	0	0
c31	Agaricomycetes	*Trechispora hymenocystis* isolate 362 (AF347090)	0	97	368	1.0	0	0
c29	Agaricomycetes	Cf. *Phlebia* sp. CBS 118.16 (AY271810)	4e^−110^	86	444	0.7	0.5	0
c46	Agaricomycetes	*Mycena galopus* strain M01 (EU924771)	0	93	478	0.4	0	0
c56	Agaricomycetes	*Russula* sp. MHM208 (EU569277)	0	97	657	0.4	0	0
c72	Agaricomycetes	*Scleroderma citrinum* isolate AWW212 (EU718119)	0	97	598	0.3	0	0
c73	Agaricomycetes	*Tomentella sublilacina* voucher KHL8457 (AF272929)	0	97	568	0.3	0	0
c12	Agaricomycetes	*Cortinarius* sp. Bear7 (FJ039690)	2e^−177^	87	499	0.3	0.5	0
c49	Agaricomycetes	*Lactarius corrugis* voucher PC BB2004–256 (EU598154)	0	95	578	0	1.0	0
c55	Agaricomycetes	*Lactarius camphoratus* voucher JMP0039 (EU819480)	0	95	732	0	1.0	0
Chytridomycota								
c77	Chytridomycota	Chytridiaceae sp. KTP-2008 (FJ214803)	0	92	580	0.4	0	0
Zygomycota								
c54	Zygomycota	*Mortierella humilis* (AJ878778)	0	99	620	0.4	0	0
Unknown Fungi								
c68	unknown	Uncultured fungus (AM260905)	0	91	487	2.0	0	0
c70	unknown	Uncultured fungus (AM260932)	4e^−41^	90	263	0.9	0	0
c65	unknown	Uncultured fungus (AM260932)	2e^−83^	78	322	0.7	1.8	0
c71	unknown	Uncultured fungus clone 6S2.13.F05 (EF619896)	1e^−55^	92	146	0.4	0	0
c75	unknown	Chytridiales sp. JEL187 (AY997035)	3e^−173^	95	170	0.4	0	0
c66	unknown	Uncultured fungus clone IH_Tag102_2534 (EU292507)	5e^−173^	88	460	0	1.1	0
c67	unknown	Ectomycorrhizal root tip 93-sepA_Ny1.EB-23.5 (AF476985)	0	96	453	0	12.5	0
c69	unknown	Uncultured fungus (AM260905)	0	90	482	0	2.7	0

### Relative frequency of fungal taxa

Our collection of cultured fungi was dominated by c32 and c80, related to *O. maius* and a *Cryptosporiopsis* sp., respectively, which were observed from all four of the sampling plots. The remaining taxa were each observed from only one sampling plot (Table 1). In the clone collection, the most frequent fungal ITS type, c1 (related to *R. ericae*) accounted for 12% and 16% of the sampled clones in the O and A horizons, respectively (Table 1). The four next most frequent taxa included c13, c74, c2, and c6 from the Sebacinales, Helotiales, and Chaetothyriales.

### Richness of ERM root fungi by soil horizon

**Figure 1:**
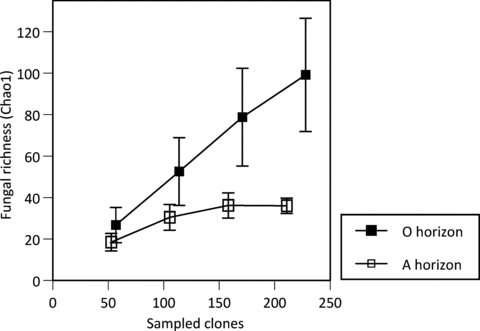
Taxa accumulation curves for ericoid mycorrhizal (ERM) root fungi in the O or A soil horizon. Chao1 richness values (and standard deviations) estimated from 1000 bootstrap replicates of the observed richness of internal transcribed spacer (ITS) sequence types from each sampling plot.

We observed greater fungal richness on ERM roots in the O horizon (48 ITS types) than in the A horizon (23 ITS types). Since fungal ITS clones were not evenly sampled between horizons (228 vs. 124 in the O and A horizon, respectively), we generated taxa richness curves using plot-level data revealing a significantly greater rate of taxa accumulation in the O versus A soil horizon (Fig. 1).

### Ascomycete MCO sequences

**Figure 2:**
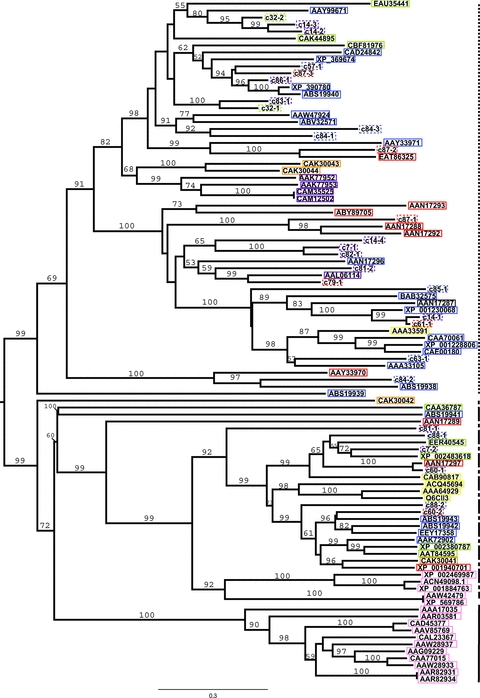
Maximum likelihood tree of multicopper oxidase amino acid sequences generated using PhyML (WAG substitution model; 1000 bootstrap replicates). Previously submitted sequences used in the alignment are identified by their accession number; sequences generated in this study are identified in Table 2. Phylogenetic assignment of each sequence is indicated by a solid- (known sequences) or dashed-lined (clone sequences from this study) box; Sordariomycetes (blue), Dothideomycetes (red), Saccharomycetes (yellow), Eurotiomycetes (green), Leotiomycetes (purple), Pezizomycetes (orange), Agaricomycetes (pink), and unclassified Ascomycota (black). Values at nodes correspond to bootstrap support in %, only values >50% are shown. Vertical lines mark three clades: dotted line = ascomycete laccases; dashed line = ferroxidases; solid line = basidiomycete laccases. Scale bar: substitutions per site.

We observed 28 unique sequences (from the *lcc2* region) from the 17 cultures of ascomycetes (Table 2). Each taxon contained one to four MCO sequence types; the only MCO sequence we obtained from isolate c80 was substantially shorter than the others and was excluded from phylogenetic analysis. MCO gene sequences were diverse and possessed moderate to strong amino acid sequence similarities (55–85%) with previously identified MCO sequences of ascomycetes. Two fungal taxa (c14–1 and c61–1) possessed nearly identical protein sequences (90%), yet were classified in the Leotiomycetes and Sordariomycetes, respectively, based on their ITS sequences. In addition, c60–1 (Sordariomycetes) was 85% similar to a previously published MCO sequence from *Pleospora spartinae* (strain SAP146), a saprotroph in the Dothidiomycetes (Lyons et al. 2003). From maximum likelihood analysis, our MCO sequences of ERM root fungi clustered into two main clades, and all the sequences of our analysis formed three clades (Fig. 2). Six MCO sequences from our study grouped in the ferroxidase clade and 22 grouped in the laccase clade; three taxa contained sequences in both clades.

**Table 2:** Multicopper oxidase gene sequence types and their closest BLASTX matches from cultured ascomycetes from *R. maximum* roots

MCO type	Closest BLASTX gene product (% identity/% similarity)	Length (nt)	*E*-value	Closest BLAST match (ITS) from Table 1
c7_1	Laccase-3, *Paracoccidioides brasiliensis* Pb01 (47/61)	930	1e^−69^	*Phialophora finlandia* strain CBS 444.86 (AF486119)
c7_2	Putative ferrooxidoreductase Fet3, *Talaromyces stipitatus* ATCC 10500 (70/84)	927	1e^−129^	
c14_1	Laccase I, *Chaetomium thermophilum* var. *thermophilum* (70/79)	925	7e^−123^	*Dermea viburni* (AF141163)
c14_2	Laccase 3, *Cryphonectria parasitica* (60/72)	904	2e^−100^	
c14_3	Laccase 3, *Cryphonectria parasitica* (66/79)	915	2e^−108^	
c14_4	Laccase, *Botryotinia fuckeliana* (44/61)	907	1e^−36^	
c32_1	Lcc4, *Fusarium oxysporum* (55/73)	905	7e^−84^	*Oidiodendron maius* strain UAMH 8921 (AF062800)
c32_2	laccase 3, *Cryphonectria parasitica* (52/68)	855	7e^−67^	
c57_1	Lcc4, *Fusarium oxysporum* (68/82)	898	4e^−110^	*Hypoxylon perforatum* isolate M4 (FJ464593)
c60_1	Laccase, *Pleospora spartinae* (84/85)	859	3e^−133^	
c60_2	Lcc3, *Fusarium oxysporum* (55/66)	933	9e^−89^	*Chloridium virescens* strain ICMP15193 (EF029220)
c61_1	Laccase I, *Chaetomium thermophilum* var*. thermophilum* (63/72)	909	1e^−103^	CSP279468 *Chaetomium* sp. 6/97–38 (AJ279468)
c63_1	Laccase, *Melanocarpus albomyces* (57/71)	916	1e^−99^	*Xylaria persicaria* strain F-165,174 (AY909022)
c81_1	Iron transport multicopper oxidase FET3, *Microsporumcanis* CBS 113480 (47/64)	772	2e^−57^	*Articulospora tetracladia* strain F-4494 (EU998918)
c81_2	Laccase-3, *Paracoccidioides brasiliensis* Pb01 (54/66)	926	4e^−51^	
c82_1	Laccase-3, *Paracoccidioides brasiliensis* Pb01 (45/60)	937	3e^−57^	*Phialophora finlandia* strain CBS 444.86 (AF486119)
c83_1	Lcc4, *Fusarium oxysporum* (67/78)	914	5e^−85^	*Fulvoflamma eucalypti* strain CPC 11243 (DQ195779)
c84_1	Laccase, *Xylaria polymorpha* (53/69)	897	5e^−87^	*Monochaetia camelliae* (AF377286)
c84_3	Laccase, *Xylaria polymorpha* (48/63)	910	1e^−68^	
c85_1	Laccase, *Gaeumannomyces graminis* var. *tritici* (50/69)	939	8e^−88^	*Glomerella acutata* strain DAOM214992 (EU400154)
c86_1	Lcc4, *Fusarium oxysporum* (67/78)	918	4e^−103^	*Pilidium acerinum* voucher BPI 843555 (AY487091)
c87_2	Laccase, *Gaeumannomyces graminis* var. *graminis* (36/55)	868	2e^−41^	*Massarina corticola* (AF383957)
c87_3	Lcc4, *Fusarium oxysporum* (67/78)	905	4e^−126^	
c88_1	Putative ferrooxidoreductase Fet3, *Talaromyces stipitatus* ATCC 10500 (58/70)	934	2e^−97^	*Ophiostoma dentifundum* strain CMW13016 (AY495434)

## Discussion

A rich and taxonomically diverse fungal community associates with ERM roots of *R. maximum*. Our findings contribute to a growing body of evidence from North America, Australia, and Europe, that ERM roots associate with fungal assemblages that are richer than what was previously observed using culture-based methods (Allen et al. 2003, Bougoure and Cairney 2005a, b; Bougoure et al. 2007; Selosse et al. 2007). Not only did we identify the classical ERM symbionts (e.g., *R. ericae* and *O. maius*; Read et al. 2004), we also observed several putative ERM symbionts, as well as documented ECM symbionts and saprotrophs. These findings on the broad composition of ERM root-fungi raise new questions about the functional diversity of these fungal communities.

We observed several taxa with strong identities to previously observed ERM-root-fungi. A *Cryptosporiopsis* taxon represented 10% of our culture collection and possessed strong similarity to *Cryptosporiopsis ericae*. This genus contains common root endophytes of oak and ericaceous plants in Europe (Sigler et al. 2005 and references therein), but has been reported as an infrequent taxon in ERM hosts of North America and Asia (Allen et al. 2003; Sigler et al. 2005; Zhang et al. 2009). We observed four taxa from the Chaetothyriales (Eurotiomycetes), together representing 25% and 3% of the sampled clones in the O and A horizons, respectively. Fungi from the Chaetothyriales have been observed on ERM roots in western Canada (Allen et al. 2003), Italy (Bergero et al. 2003), Australia (Bougoure and Cairney 2005b), and Scotland (Bougoure et al. 2007), and while they colonize ERM root cortical cells in resynthesis trials, their effect on plant growth and nutrition is unknown (Bergero et al. 2000; Allen et al. 2003). Another group of putative ERM symbionts belongs to the newly defined order Sebacinales (Agaricomycotina), which are commonly cloned from roots of many plant species but have not been successfully cultured (Allen et al. 2003; Selosse et al. 2007; Weiβ et al. 2011). Sebacinalean fungi were present in 31% of root samples collected from ericaceous plants worldwide (Selosse et al. 2007), accounted for nearly 60% of fungal clones from *G. shallon* ERM roots (Allen et al. 2003) and accounted for 19% and 6% of fungal ITS clones from *R. maximum* ERM roots in the O and A horizon, respectively (this study). Therefore, many similarities in the composition of ERM root fungi have emerged from the handful of studies utilizing molecular methods in spite of the fact they have been conducted from disparate parts of the globe.

We also observed several taxa that are closely related to ECM fungi including *Cenococcum geophilum* and others in *Elaphomyces*, *Tomentella*, *Scleroderma* and Russulaceae. Previous research has described the colonization of ERM roots by ECM fungi (Smith et al. 1995), the appearance of fungal mantles on ERM roots of *R. maximum* (Dighton and Coleman 1992), as well as simultaneous colonization of ERM and ECM roots by a single mycelium of *Cadophora* sp. (Villareal–Ruiz et al. 2004). Similar to the observations of Bougoure et al. (2007), we found that ECM taxa accounted for one-third of the sampled root-clones belonging to the Basidiomycota, and in our study, ascomycetous and basidiomycetous ECM taxa together represented 9% and 3% of the observed root fungi in the O and A horizon. Although it is unknown if ECM fungi play an important functional role when associating with ERM roots, the repeated documentation of ECM fungal taxa on ERM roots certainly warrants further investigation.

We compared the ERM fungal community between the organic and upper mineral soil horizons. Consistent with our expectation, we observed greater fungal richness on roots in organic than in mineral soil horizons, suggesting that ERM root fungi are favored in organic substrates. The relative frequencies of some fungal groups indicate a preference for one horizon over the other. For example, taxa from the Basidiomycota were sampled more frequently from the organic than from the mineral soil horizon (28% vs. 9%; paired *t*-test *p* = 0.03), while taxa from Leotiomycetes tended to be more frequent in the mineral than the organic soil horizon (61% vs. 29%; paired *t*-test *p* = 0.09). Interestingly, taxa with strong identities to *R. ericae* and *O. maius*, well-described ERM symbionts, were cloned from both soil horizons at frequencies that were not significantly different (paired *t*-tests, *p* = 0.70 and *p* = 0.74, respectively). Since several known saprotrophic fungi were observed on ERM roots in our study (e.g., c48, c57, c63, c81, and c83, Sánchez–Ballesteros et al. 2000; Nikolcheva et al. 2003; Crous et al. 2006; Fernández et al. 2006; Han and Shin 2007), it is possible that we amplified fungi from soil particles despite our attempts to remove debris adhering to the sampled roots. Alternatively, if ERM roots associate with a broad spectrum of soil fungi, the patterns of fungal richness we observed in ERM roots may be driven by the richness of soil fungi, which tends to decline from organic to mineral horizons (O’Brien et al. 2005). ECM fungi, saprotrophs and endophytes have been repeatedly documented to colonize ERM roots (Allen et al. 2003; Bougoure & Cairney 2005; Selosse et al. 2007; Vohník et al. 2007; Zhang et al. 2009) providing further support that ERM roots associate with a variety of soil fungi. These root–fungal associations may not all represent mycorrhizal symbioses in a strict sense, but they may still provide indirect benefits to the plant host by promoting decomposition and the release of nutrients from organic matter.

From our culture collection of ERM-root-ascomycetes, we isolated at least one, and as many as four, unique MCO sequence types per taxon. Multiple copies of MCOs are common among fungi and may be the result of initial duplication events followed by evolution (Valderrama et al. 2003). MCOs perform a variety of important functions including lignin degradation (Leonowicz et al. 2001), tissue development, and melanin biosynthesis (Hoegger et al. 2006). From our phylogenetic analysis, MCOs from ERM root-associating ascomycetes did not group strictly by taxonomy, but rather, they clustered in two distinct clades representing ferroxidases and laccases. Some ferroxidases are membrane-bound proteins involved in iron uptake and cellular homeostasis (De Silva et al. 1995); however, a number of the sequences in this clade are observed from genomic DNA and are not functionally defined. Among MCOs, laccases are likely to be functionally important for decomposition processes in soils and to aid in releasing nutrients from organic matter (Leonowicz et al. 2001; Kellner et al. 2007).

The laccase clade of our phylogenetic tree contains sequences coding for laccase from ascomycetous plant pathogens and terrestrial and aquatic saprotrophs from previous studies. Twenty-two MCO sequences from 13 of our cultured ERM-root-fungi grouped closely among these characterized laccase sequences. We observed two copies of MCO gene sequences from *O. maius* (c32 in our study), one of which grouped closely to a tannic acid inducible laccase from *Cryphonectria parasitica* (AAY9971; Chung et al. 2008). Cultures of *O. maius* have been verified to oxidize polyaromatic substrates (Bending and Read 1997) and produce extracellular polyphenol oxidases (Thormann et al. 2002). Other well-studied sequences from the laccase clade represent extracellular laccases from the saprotroph *Verpa conica* (CAK30043) and the ECM fungus *Morchella conica* (CAK30044) whose expressions were inducible by a phenolic compound (Kellner et al. 2007). Whether any of the diverse ERM root-fungi observed in our study are prolific producers of extracellular laccases is an unanswered and critical question. Although the function of these MCO gene sequences is not verified, they provide a means for further exploration of MCO diversity and expression among ERM root-fungi. This genetic approach could be useful for broadening our view of how ERM root-fungi, particularly those that are not classical symbionts, provide indirect benefits to host plants and contribute to biogeochemical cycling in terrestrial ecosystems.

ERM roots of *R. maximum* associate with a taxonomically and ecologically diverse assemblage of fungi. To date, only two verified ERM symbionts (or species complexes; *R. ericae* and *O. maius*) are known, and representatives of both groups were sampled in this study. We also observed a number of putative ERM symbionts, ECM symbionts, and saprotrophs that have been repeatedly observed on ERM roots from around the world (Allen et al. 2003, Bougoure and Cairney 2005a, b; Selosse et al. 2007; Bougoure et al. 2007). The significance of many of these relationships for the host plant remains largely unexplored. Since the saprotrophic ability of root-fungi is presumed to be a more crucial functional trait for ERM hosts than for ECM and AM hosts (Read et al. 2004), testing the ability of ERM root-fungi to produce extracellular enzymes may provide a mechanistic approach to understanding how they contribute to decomposition. We observed a number of MCO gene sequences from ascomycetous ERM root-fungi that group closely with known laccases. The ability of ERM root-fungi to produce these oxidative enzymes may be a key mechanism for ERM plants to acquire nutrients from the organic-rich soils in which they persist.
